# Renal Prognosis and Related Risk Factors for Henoch-Schönlein Purpura Nephritis: A Chinese Adult Patient Cohort

**DOI:** 10.1038/s41598-018-23638-2

**Published:** 2018-04-03

**Authors:** Xiao Huang, Xiaomei Wu, Weibo Le, Yaxin Hao, Jing Wu, Caihong Zeng, Zhihong Liu, Zheng Tang

**Affiliations:** 1 National Clinical Research Center of Kidney Diseases, Jinling Hospital, Nanjing University School of Medicine, Nanjing, P. R. China; 2 Department of Endocrine, Nanjing Pukou Central Hospital, Nanjing Medical University, Nanjing, P. R. China

## Abstract

This study investigated the clinicopathological characteristics of Henoch-Schönlein purpura nephritis (HSPN) in Chinese adult patients and analyzed the renal outcomes and prognostic risk factors for progression to end-stage renal disease (ESRD). Adult patients who had biopsy-proven HSPN were studied. Their clinicopathological data, renal prognoses and related risk factors were assessed. A total of 698 patients were studied, including 363 men (52.0%) and 335 women (48.0%). Most of the patients had hematuria (85.8%) and/or proteinuria (82.1%). During a median follow-up of 54.0 months, 32 patients (4.6%) progressed to ESRD. The 5- and 10-year cumulative renal survival rates from ESRD were 96.4% and 88.6%, respectively. Baseline urinary protein, renal insufficiency, glomerular sclerosis and tubular atrophy/interstitial fibrosis were independent predictors of renal outcomes. Both the time-average mean arterial pressure and proteinuria during follow-up also influenced the renal prognosis. The patients with a time-average proteinuria <0.4 g/day had the lowest rates of ESRD or a 50% decline in renal function. In conclusion, identifying of clinical and histological prognostic factors may permit the prediction of renal outcomes. The optimal goal of therapy for HSPN patients may be to lower proteinuria to <0.4 g/day and control hypertension to achieve an ideal renal outcome.

## Introduction

Henoch-Schönlein purpura (HSP) affects people of all ages, but 90% of cases occur in those less than 10 years of age^1^. The course of HSP is generally benign and self-limited in children^2^. Kidney involvement occurs in approximately 30–50% of children with HSP^3,4^. Microscopic hematuria is the most common finding. Macroscopic hematuria, proteinuria, nephrotic syndrome and reduced kidney function are also common manifestations^1,5,6^. Henoch-Schönlein purpura nephritis (HSPN) has increasingly been identified as a major cause of chronic renal failure in the pediatric patient group^7^, with approximately 5–15% of children with HSPN progressing to chronic renal failure^8^.

Adults with HSP present with renal involvement (49–83%) of variable significance^9^. As shown in previous studies, the clinical presentations and renal outcomes can be more severe in adults than in children with HSPN^10^, with an estimated 25% to 30% risk of progression to chronic renal insufficiency^11^. Considerably fewer reports have investigated the renal outcomes of HSPN in adults than those in children. In addition, the study sample sizes are relatively small^12,13^, and the follow-up time is not sufficient to predict renal prognosis accurately^14,15^.

As a result, we retrospectively analyzed adult patients who suffered from HSP and had biopsy-proven renal involvement in our center. The aim of our study was to describe the clinical, laboratory and histological characteristics of HSPN in a Chinese adult patient cohort. Long-term renal survival and prognostic factors were also evaluated.

## Materials and Methods

Adult patients (age at biopsy ≥18 years old) with biopsy-proven HSPN who were treated in Nanjing Jinling Hospital between January 2003 and December 2013 were reviewed. HSPN was diagnosed when hematuria or proteinuria was associated with a palpable purpuric eruption and/or abdominal and/or joint pain^16,17^. Patients who suffered from diabetes mellitus, chronic liver disease, acute interstitial nephritis, malignancy and other autoimmune disorders were excluded. Patients without complete clinicopathological data and with <12 months of follow-up were also excluded except for patients who reached end-stage renal disease (ESRD) within 12 months. All follow-up data were updated to November 2016. This study followed the tenets of the Declaration of Helsinki.

General characteristics were recorded, including gender, age at onset and at biopsy, duration and stimulus. Clinical features, including the presence of hypertension, renal involvement and extra-renal manifestations, were assessed. Blood and urine samples were obtained from individual patients at the time of biopsy for routine testing. The laboratory data included hematuria, 24-hour urinary protein, hemoglobin, serum creatinine, urea nitrogen, serum uric acid, serum albumin and cholesterol. Pathological changes were evaluated, including glomerular sclerosis, segmental sclerosis, crescents, glomerulus-Bowman’s capsule adhesion, capillary necrosis and tubular atrophy/interstitial fibrosis. Tubular atrophy/interstitial fibrosis was semi-quantitatively graded as none, mild, moderate and severe^18^. Immunofluorescence for immunoglobulin G (IgG), immunoglobulin A (IgA), immunoglobulin M (IgM), complement 3 (C3), and complement 1q (C1q) deposits was semi-quantitatively graded from 0 to 3 according to the fluorescence intensity^19^.

The renal survival time was calculated from the biopsy to the last follow-up. The time-average mean arterial pressure (TA-MAP), time-average proteinuria (TA-P) and microscopic hematuria (TA-RBC) were calculated. Two clinical outcomes were defined. The primary end point was ESRD, and the secondary end point was a composite of ESRD or a 50% reduction in renal function.

Hypertension was diagnosed according to the standards recommended by the World Health Organization Expert Committee^12^. Microscopic hematuria was measured as counts of erythrocytes per millimeter of urinary sediment and was between 10 and 1000 × 10^4^/mL. Macroscopic hematuria was defined as >1000 × 10^4^/mL. Proteinuria was defined as proteinuria >0.4 g/day. Nephrotic syndrome was defined as plasma albumin <35 g/L and proteinuria >3.5 g/day; patients with albumin <30 g/L were also included in this category if their proteinuria was between 3.0 and 3.5 g/day. The estimated glomerular filtration rate (eGFR) was calculated using the chronic kidney disease epidemiology collaboration (CKD-EPI) formula. Renal insufficiency was defined as an eGFR <60 mL/min/1.73 m^2^. ESRD was defined as an eGFR <15 mL/min/1.73 m^2^, initiation of dialysis or transplantation for over three months. Hypoproteinemia and hypercholesterolemia were defined as serum albumin <30 g/L and cholesterol >6.2 mmol/L, respectively. TA-MAP was evaluated as the ratio of the area under the MAP curve during follow-up. TA-P and TA-RBC were calculated using the same method^20^.

### Statistical Analysis

The SPSS 18.0 software (SPSS, Chicago, IL, USA) was used for the statistical analysis. Normally distributed variables were expressed as the means ± SDs. Non-parametric variables are expressed as medians and ranges. Categorical variables were expressed as percentages. Renal survival was estimated using the Kaplan–Meier method. The relationship between the parameters and renal survival was assessed using Cox regression. Receiver operating characteristic (ROC) curves were drawn for variables to determine the optimal cut-off values to predict an endpoint. All P-values were two-tailed, and values <0.05 were considered statistically significant.

### Data availability statement

The datasets analyzed during the current study are available from the corresponding author upon reasonable request.

### Statement of Ethics

The protocol followed in the present study was approved by the Jinling Hospital Ethics Committee on Human Experimentation. Due to the retrospective nature of the study, written informed consent for participation in the study was waived.

## Results

### Demographic Characteristics of the Adult HSPN Patients at Biopsy

A total of six hundred and ninety-eight patients with adult HSPN were included with 363 men (52.0%) and 335 women (48.0%). The median ages at onset and renal biopsy were 25.0 and 29.0 years, respectively. The intervals between onset and renal biopsy were skewed (median 11.5 months, interquartile range 2.0–48.0 months). The common stimuli associating with the initial clinical manifestations of HSP were infection (11.6%), drugs (7.4%) and food (19.2%); however, 57.9% of the patients had no clear reason for HSP induction (Table 1).

**Table 1:** Demographic characteristics and clinical features of the adult HSPN patients at biopsy.

Items	Values (n = 698)	Items	Values (n = 698)
Male, n (%)	363 (52.0)	Renal involvement	
Age at onset, years	25.0 (19.0–37.0)	Previous macroscopic hematuria, n (%)	133 (19.1)
Age at biopsy, years	29.0 (22.0–39.0)	Recurrent macroscopic hematuria, n (%)	29 (4.2)
Duration, months	11.5 (2.0–48.0)	Macroscopic hematuria, n (%)	52 (7.4)
Purpura duration, months	9.0 (2.0–48.0)	Microscopic hematuria, n (%)	547 (78.4)
Renal duration, months	5.0 (1.0–24.0)	Proteinuria, n (%)	573 (82.1)
Inducement		Nephrotic syndrome, n (%)	69 (9.9)
Infection, n (%)	81 (11.6)	Renal insufficiency, n (%)	51 (7.3)
Drug, n (%)	52 (7.4)	Extrarenal manifestation	
Food, n (%)	134 (19.2)	Purpura, n (%)	698 (100)
Seafood, n (%)	105 (15.0)	Gastrointestinal symptoms, n (%)	191 (27.4)
Other, n (%)	55 (7.9)	Presence of arthritis, n (%)	207 (29.7)
No clear inducement, n (%)	404 (57.9)	Hypertension, n (%)	147 (21.1)

### Clinical and Laboratory Data for the Adult HSPN Patients at Biopsy

All the enrolled patients had manifestations of renal involvement and purpura. Gastrointestinal symptoms and arthritis were present in 27.4% and 29.7% of the patients, respectively. The renal involvement was variable. Macroscopic hematuria occurred in 7.4% of the patients, and 78.4% of the patients had microscopic hematuria at biopsy. The incidence of proteinuria was 82.1%, and 9.9% of the patients presented with nephrotic syndrome. In addition, 7.3% of the patients suffered from renal insufficiency. Hypertension occurred in 21.1% of the patients (Table 1).

The median proteinuria at biopsy was 0.88 g/day (interquartile range 0.51–1.79 g/day). The median serum creatinine and blood urea nitrogen levels were 0.77 mg/dL (interquartile range 0.63–0.97 mg/dL) and 13.3 mg/dL (interquartile range 10.6–17.0 mg/dL), respectively. The mean uric acid levels were 333.1 ± 89.0 µmol/L. A total of 65 patients (9.3%) suffered from hypoproteinemia, and 155 patients (22.2%) had hypercholesterolemia (Table 2).

**Table 2:** Laboratory characteristics and renal pathological findings of the adult HSPN patients at biopsy

Items	Values (n = 698)	Items	Values (n = 698)
**Laboratory data**		**Light microscopy**	
Hematuria (×10^4^/mL)	90 (30–250)	Glomerular sclerosis (%)	5.0 (0–13.7)
Urinary protein (g/day)	0.88 (0.51–1.79)	Glomerular sclerosis >10%, n (%)	192 (27.5)
<0.4 g/day, n (%)	125 (17.9)	Segmental sclerosis (%)	0 (0–7.1)
0.4–1.0 g/day, n (%)	256 (36.7)	Segmental sclerosis >10%, n (%)	118 (16.9)
1.0–3.5 g/day, n (%)	250 (35.8)	Crescents (%)	5.0 (0–12.6)
>3.5 g/day, n (%)	67 (9.6)	Crescents ≥25%, n (%)	60 (8.6)
Serum albumin (g/L)	39.5 (35.4–42.8)	Glomerulus-Bowman’s capsule adhesion, n (%)	331 (47.4)
Hypoproteinemia, n (%)	65 (9.3)	Capillary necrosis, n (%)	166 (23.8)
Hemoglobin (g/dL)	13.0 ± 1.7	Moderate and severe tubular atrophy/interstitial fibrosis, n (%)	82 (11.7)
Serum creatinine (mg/dL)	0.77 (0.63–0.97)	**Immunofluorescence**	
Blood urea nitrogen (mg/dL)	13.3 (10.6–17.0)	IgG, n (%)	109 (15.6)
Serum uric acid (µmol/L)	333.1 ± 89.0	IgM, n (%)	252 (36.1)
Hypercholesterolemia, n (%)	155 (22.2)	C3, n (%)	607 (87.0)
		C1q, n (%)	16 (2.3)

IgG, immunoglobulin G; IgM, immunoglobulin M; C3, complement 3; C1q complement 1q.

### Renal Pathological Findings of the Adult HSPN Patients at Biopsy

The renal pathological evaluation focused on renal components including the glomeruli, tubules and interstitium. The median percentage of glomerular sclerosis was 5.0% (interquartile range 0–13.7%). Glomerular sclerosis of >10% was seen in 192 patients (27.5%). Segmental sclerosis was also observed and involved more than 10% of the glomeruli in 118 patients (16.9%). The median proportion of crescents was 5.0% (interquartile range 0–12.6%), and 60 patients (8.6%) had more than 25% crescents. Bowman’s capsule adhesion was present in 47.4% of the patients, and the incidence of capillary necrosis was 23.8%. A total of 82 patients (11.7%) suffered from moderate and severe lesion of tubular atrophy/interstitial fibrosis lesions. The occurrence of immune complex deposits was as follows: 15.6% with IgG, 36.1% with IgM, 87.0% with C3, and 2.3% with C1q. (Table 2).

### Follow-up and Renal Outcomes of the Adult HSPN Patients

The therapies after biopsy were analyzed and shown in Table 3. A total of 166 patients (23.8%) received methylprednisolone pulse treatment and 474 patients (67.9%) were treated with oral prednisone/methylprednisolone. Corticosteroid combined with other immunosuppressive agents was administered as follows: 272 patients (39.0%) with prednisone and tripterysium glycosides, 98 patients (14.0%) with prednisone and mycophenolate mofetil, and 14 patients (2.0%) with prednisone and leflunomide.

**Table 3:** Treatment and renal outcomes of the adult HSPN patients

Items	Values (n = 698)
**Treatment**	
Methylprednisolone pulse treatment, n (%)	166 (23.8)
Prednisone, n (%)	474 (67.9)
Prednisone + tripterysium glycosides, n (%)	272 (39.0)
Prednisone + leflunomide, n (%)	14 (2.0)
Prednisone + mycophenolate mofetil, n (%)	98 (14.0)
**Follow-up**	
Follow-up from biopsy, months	54.0 (34.0–78.0)
TA-P (g/day)	0.45 (0.30–0.74)
<0.4 g/day, n (%)	295 (42.3)
0.4–1.0 g/day, n (%)	290 (41.5)
>1.0 g/day, n (%)	113 (16.2)
TA-RBC (×10^4^/mL)	35 (14–87)
TA-MAP (mmHg)	91.0 (85.0–97.0)
**Outcomes**	
ESRD, n (%)	32 (4.6)
ESRD or a 50% decline in renal function, n (%)	53 (7.6)

TA-P time-average proteinuria; TA-RBC time-average microscopic hematuria;

TA-MAP time-average mean arterial pressure; ESRD end-stage renal disease.

**Figure 1:**
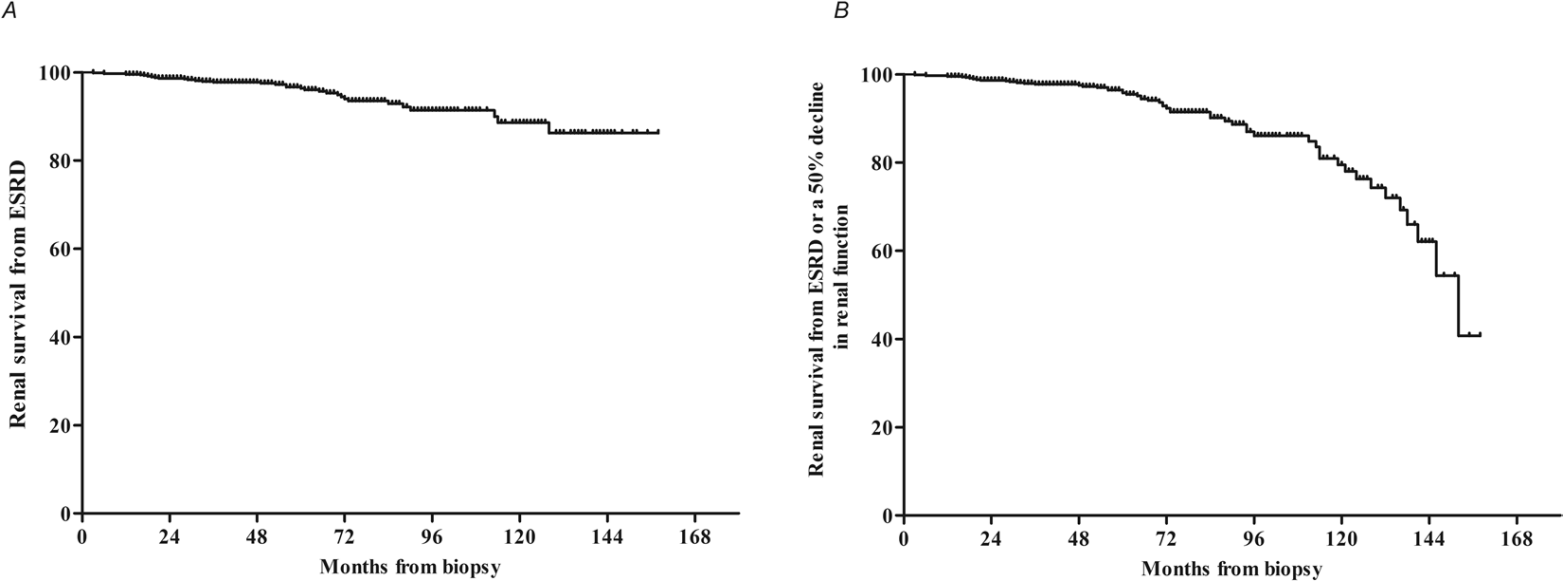
The Kaplan-Meier analysis of renal survival from ESRD (**A**) and ESRD or a 50% decline in renal function (**B**) for patients with HSPN.

During a median follow-up of 54.0 months (interquartile range, 34.0–78.0 months), the median TA-P measurement of the enrolled adult HSPN patients was 0.45 g/day (interquartile range, 0.30–0.74 g/day). The median TA-MAP value during follow-up was 91.0 mmHg (interquartile range, 85.0–97.0 mmHg). Ultimately, 32 patients (4.6%) developed ESRD, and 53 patients (7.6%) achieved a composite of ESRD or a 50% decline in renal function (Table 3). The 5- and 10-year cumulative renal survival rates from ESRD after renal biopsy as calculated using the Kaplan-Meier methods were 96.4% [95% confidence interval (CI), 94.6–98.2%] and 88.6% (95% CI, 83.5–93.7%), respectively (Fig. 1A). The 5- and 10-year cumulative rates for the composite of ESRD or a 50% decline in renal function were 95.8% (95% CI, 94.0–97.6%) and 79.6% (95% CI, 72.5–86.6%), respectively (Fig. 1B).

### Risk Factors at Biopsy and During Follow-up of the Adult HSPN Patients

Hypertension, urinary protein >1.0 g/day, an eGFR <60 mL/min/1.73 m^2^, and hypoproteinemia predicted an increased risk of ESRD in the univariate analysis. Renal pathological parameters, including glomerular sclerosis >10%, segmental sclerosis >10%, and moderate and severe tubular atrophy/interstitial fibrosis, were also significantly associated with disease progression. Parameters that were significant in the univariate analysis were further considered in a multivariate Cox regression model. This model confirmed that urinary protein >1.0 g/day [hazard ratio (HR) 2.486, P = 0.031], an eGFR <60 mL/min/1.73 m^2^ (HR 5.344, P < 0.001), glomerular sclerosis >10% (HR 2.397, P = 0.044) and moderate and severe tubular atrophy/interstitial fibrosis (HR 5.239, P < 0.001) were independent predictors of renal outcomes, whereas the other parameters failed to reach statistical significance (Table 4). As shown in Table 5, TA-P, TA-MAP during follow-up, baseline hypertension, baseline proteinuria and an eGFR <60 mL/min/1.73 m^2^ at biopsy were analyzed in the multivariate Cox model. This analysis confirmed that both TA-P (HR 2.193, P < 0.001) and TA-MAP (HR 1.043, P = 0.041) were independent predictors of renal survival from ESRD. The multivariate Cox analysis also revealed that interval increase of 100 mg/day in TA-P increased the risk of ESRD by 1.082 (P < 0.001).

**Table 4:** Factors at biopsy influencing renal survival from ESRD based on univariate and multivariate Cox Regression analyses.

Items	Univariate		Multivariate	
	HR (95% CI)	P	HR (95% CI)	P
Hypertension	2.971 (1.477–5.975)	P = 0.002^**^	—	—
Urinary protein >1.0 g/day	3.693 (1.659–8.222)	P = 0.001^**^	2.486 (1.087–5.685)	P = 0.031^*^
eGFR <60 mL/min/1.73 m^2^	15.556 (7.501–32.262)	P < 0.001^**^	5.344 (2.297–12.433)	P < 0.001^**^
Hypoproteinemia	3.700 (1.709–8.011)	P = 0.001^**^	—	—
Crescents ≥25%	2.081 (0.800–5.411)	P = 0.133	—	—
Glomerular sclerosis >10%	5.778 (2.732–12.219)	P < 0.001^**^	2.397 (1.026–5.603)	P = 0.044^*^
Segmental sclerosis >10%	3.224 (1.572–6.612)	P = 0.001^**^	—	—
Moderate and severe tubular atrophy/interstitial fibrosis	12.682 (6.235–25.795)	P < 0.001^**^	5.239 (2.274–12.071)	P < 0.001^**^

eGFR estimated glomerular filtration rate; HR hazard ratio; CI confidence interval.

^*^P < 0.05, ^**^P < 0.01.

**Table 5:** Factors during follow-up influencing renal survival from ESRD based on univariate and multivariate Cox regression analyses.

Items	Univariate		Multivariate^#^	
	HR (95% CI)	P	HR (95% CI)	P
TA-P	2.739 (2.317–3.238)	P < 0.001^**^	2.193 (1.783–2.699)	P < 0.001^**^
TA-MAP	1.091 (1.059–1.123)	P < 0.001^**^	1.043 (1.002–1.085)	P = 0.041^*^
TA-RBC	1.001 (0.999–1.003)	P = 0.252	—	—

^#^Multivariate Cox model: multivariate with TA-P, TA-MAP, baseline hypertension, baseline proteinuria and an eGFR <60 mL/min/1.73 m^2^ at biopsy.

HR hazard ratio; CI confidence interval; TA-P time-average proteinuria; TA-RBC time-average microscopic hematuria; TA-MAP time-average mean arterial pressure.

^*^P < 0.05, ^**^P < 0.01.

**Figure 2:**
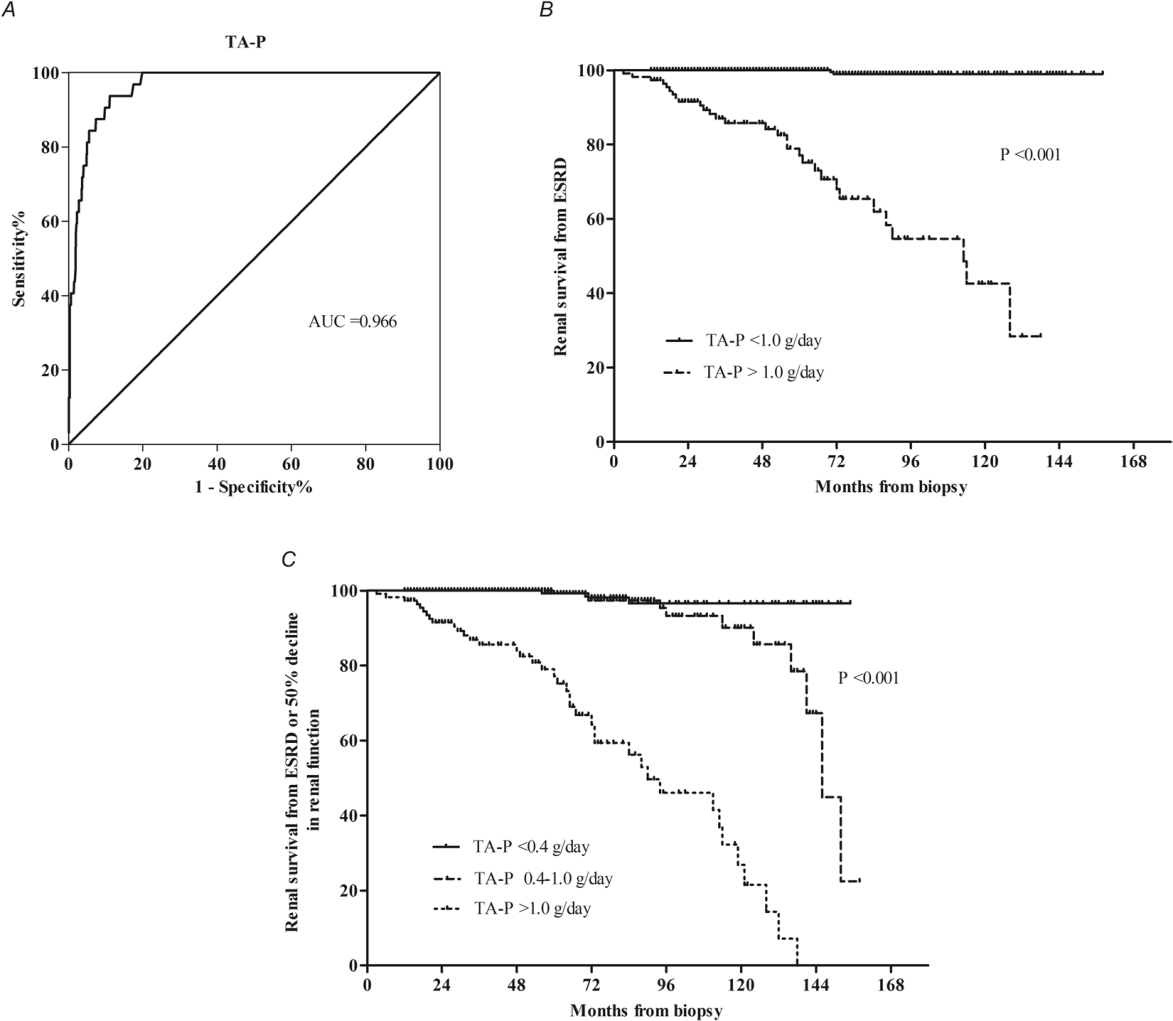
Cumulative renal survival rates stratified according to TA-P in the adult HSPN patients. (**A**) ROC analysis of TA-P; (**B**) Renal survival rates from ESRD in two groups based on TA-P >1.0 g/day and TA-P <1.0 g/day; (**C**) Renal survival rates from ESRD or a 50% decline in renal function in three groups based on TA-P >1.0 g/day, 0.4–1.0 g/day and TA-P <0.4 g/day.

The area under the ROC curve (AUC) for TA-P was 0.966. The optimal cut-off for TA-P was 1.045 g/day (sensitivity, 93.8%; specificity, 88.9%), which was estimated as 1.0 g/day (Fig. 2A). The renal survival rates from ESRD in each group are shown in Fig. 2B. We analyzed the renal outcomes based on categorical grouping of TA-P excretion by Cox regression. Multivariate analyses with TA-P, TA-MAP, baseline hypertension, baseline proteinuria and an eGFR <60 mL/min/1.73 m^2^ at biopsy revealed that the patients with TA-P >1.0 g/day were associated with a higher risk (HR 59.689, P < 0.001) of ESRD than those with TA-P <1.0 g/day, which predicted a poor renal outcome. We also set the TA-P cut-offs at 0.4 g/day and 1.0 g/day to divide the patients into three groups. The univariate and multivariate Cox analysis indicated that the patients with TA-P 0.4–1.0 g/day were not associated with a higher risk of ESRD than those with TA-P <0.4 g/day (Table 6). Moreover, the renal survival rates from ESRD or a 50% decline in renal function were significantly different among the three groups, as shown in Fig. 2C. The multivariate Cox analysis indicated that patients with both TA-P levels >1.0 g/day (HR 42.629, P < 0.001) and 0.4–1.0 g/day (HR 3.715, P = 0.045) were associated with higher risks of ESRD or a 50% decline in renal function than those with TA-P <0.4 g/day (Table 6).

**Table 6:** Renal outcomes based on categorical grouping of TA-P and TA-MAP excretion during follow-up based on univariate and multivariate Cox regression analyses.

Items	Renal survival from ESRD				Renal survival from ESRD or a 50% decline in renal function			
	Univariate (HR, 95% CI)	P	Multivariate(HR, 95% CI)^#^	P	Univariate (HR, 95% CI)	P	Multivariate (HR, 95% CI)^#^	P
TA-P								
<1.0 g/day	1.0		1.0		1.0		1.0	
>1.0 g/day	95.426 (22.779–399.764)	P < 0.001^**^	59.689 (13.947–255.458)	P < 0.001^**^	28.926 (14.347–58.320)	P < 0.001^**^	19.926 (9.559–41.534)	P < 0.001^**^
TA-P								
<0.4 g/day	1.0		1.0		1.0		1.0	
0.4–1.0 g/day	1.892 (0.171–20.865)	P = 0.603	1.766 (0.161–19.613)	P = 0.639	3.803 (1.057–13.683)	P = 0.041^*^	3.715 (1.031–13.391)	P = 0.045^*^
>1.0 g/day	90.347 (12.295–633.909)	P < 0.001^**^	53.791 (7.173–403.416)	P < 0.001^**^	62.624 (18.353–213.682)	P < 0.001^**^	42.629 (12.176–149.244)	P < 0.001^**^

^#^Multivariate Cox model: multivariate with TA-P, TA-MAP, baseline hypertension, baseline proteinuria and an eGFR <60 mL/min/1.73 m^2^ at biopsy.

ESRD end-stage renal disease; HR hazard ratio; CI confidence interval; TA-P time-average proteinuria.

^*^P < 0.05, ^**^P < 0.01.

## Discussion

Although numerous long-term renal survival analyses have been reported for pediatric HSPN patients in developed countries^8,21,22^, few studies have calculated the clinical features and long-term renal survival rates in adult HSPN patients. Data on this disease in adults are confined to small series with relatively short follow-ups^9,13,14,23–25^, especially in adult Chinese patients. In this study, we reported the epidemiology and spectrum of clinicopathological features in adult HSPN patients and identified the 10-year cumulative renal survival rate in a Chinese cohort with a larger number of patients and longer follow-up and analyzed the risk factors for ESRD in those patients.

The patients enrolled in this study had a slight male predominance. In most previous studies in both children and adults, the proportion of male patients has been higher^12,26^. However, some studies showed a female predominance^22,27^. Concerning the induction of HSP, several patients had a recent history of infection, mostly of the upper respiratory tract, but 57.9% of the patients in our cohort had no clear reason for disease induction. However, 46% of the patients in an adult population in the UK had histories of infection preceding presentation^13^. These variations in general and clinical features may be attributable to the different sample sizes, reporting standards, time frames, races and geographical areas. Due to the limitations of the study candidates, all the patients in our study had purpura and renal involvement. The other extra-renal manifestations, incidences of gastrointestinal symptoms and presence of arthritis were lower than those of the pediatric group, which was confirmed by previous reports^13,28^.

Microscopic hematuria and proteinuria were the most common renal manifestations in this study. However, hematuria failed to predict the renal prognosis. These findings are consistent with those from previous reports^23,24^. In other studies, hypertension was an independent predictor of the renal prognosis^9,13^. Hypertension did not reach statistical significance for predicting renal outcome in our analysis. However, our study demonstrated that TA-MAP during follow-up was a risk factor for predicting renal outcomes. Thus, controlled hypertension predicted good renal prognosis for patients. Moreover, approximately one-quarter of patients had reduced renal function. The multivariate analysis demonstrated that renal insufficiency was an independent predictor of the renal outcome, which was consistent with other studies^13,23^.

Regarding the renal pathology of HSPN patients, the multivariate analysis showed that glomerular sclerosis >10% was an independent predictor of the renal outcome, and other studies also found that glomerular lesions had a prognostic value^13,23^. Several acute renal pathological injury manifestations, such as crescents, glomerulus-Bowman’s capsule adhesion and capillary necrosis, failed to associate with the renal outcome. The pathological classification of HSPN in children is widely conducted according to the International study of Kidney Disease in Children (ISKDC) pathology grade, which is based in detail on the degree of mesangial proliferation and the presence of crescents. Most patients in our study had fewer than 10% crescents, which was similar to the results of other studies^9,14,29^. In a Korean HSPN cohort, 25% of the patients were within ISKDC IV and 8% were within ISKDC V with a higher proportion of crescents^30^. However, the duration between symptom onset of and the biopsy procedure was 112 (35–293) days^30^, which was shorter than the duration in our study. Therefore, duration may be an important factor that influences the proportion of crescents and glomerular and segmental sclerosis at biopsy. Moreover, the presence of crescents may not be a sign of irreversible damage in patients with HSPN and thus may not be predictive of the long-term outcome^9,30^. However, some studies investigating the predictors of outcomes in children and adults with HSPN demonstrated that crescentic nephritis was significantly related to functional decline^9,31^. In addition, chronic injury to the tubules and interstitium was significantly associated with the outcome in the multivariate analysis, which was consistent with other related studies^23,30^.

During the follow-up period in our study, 4.6% of all the patients developed ESRD. The 10-year cumulative renal survival rate from ESRD was 88.6%. Several previous studies have shown that renal survival rates in adult HSPN patients can reach 80% at 10 years^9,13,23^. In a study with thirty-seven patients in a UK population, 27% of the patients progressed to end-stage renal failure with a 72% survival rate at 5 years, a 68% at 10 years and a 46% rate at final review^13^. A French study with a 14.8-year follow-up period reported that only 11% of the patients proceeded to ESRD, and similar results were found in other populations^23,24,32^. Compared with previous studies, a lower percentage of the patients progressed to ESRD in our cohort. Thus, the sample size, race, geographical area and treatment may have influenced the results. The follow-up time was relatively short, which could lead to a better apparent prognosis.

Some independent risk factors at biopsy, such as hypertension, renal function impairment, glomerular sclerosis and tubular atrophy/interstitial fibrosis, have been associated with a poor renal prognosis in most studies^9,13,23,31^. However, not all prognostic factors are the same, and the parameters incorporated into the analysis may also matter. Moreover, variables during follow-up are seldom identified. A previous study that enrolled thirty-seven adult patients with HSPN reported that both proteinuria over 1.0 g/day and hypertension during follow-up were risk factors for ESRD^13^. Another study also reported that the risk for progression of HSPN was associated with increasing mean proteinuria levels during follow-up^9^. Our study also suggests that TA-P levels should be controlled optimally to <1.0 g/day. Moreover, we found that patients who achieved TA-P <0.4 g/day benefited more than those with TA-P between 0.4 and 1.0 g/day. Thus, the optimal goal of anti-proteinuric therapy for Chinese adult HSPN patients is to lower proteinuria <0.4 g/day during follow-up.

In conclusion, identifying of clinical and histological prognostic factors may permit the prediction of renal outcomes. The optimal goal of therapy for patients may be to lower proteinuria to <0.4 g/day and to control hypertension to predict a good renal prognosis for HSPN patients.
